# Bile acid receptor agonists in primary biliary cholangitis: Regulation of the cholangiocyte secretome and downstream T cell differentiation

**DOI:** 10.1096/fba.2018-00046

**Published:** 2019-04-22

**Authors:** Rachel E. Etherington, Benjamin J. M. Millar, Barbara A. Innes, David E. J. Jones, John A. Kirby, John G. Brain

**Affiliations:** ^1^ Institute of Cellular Medicine Newcastle University Newcastle upon Tyne UK

**Keywords:** autoimmunity, FGF19, FXR, senescence, TGR5

## Abstract

Primary biliary cholangitis (PBC) is a chronic autoimmune liver disease. Approximately 30% of patients do not respond to therapy with ursodeoxycholic acid (UDCA). Previous studies have implicated increased senescence of cholangiocytes in patients who do not respond to UDCA. This may increase the release of cytokines which drive pathogenic T cell polarization. As FXR agonists are beneficial in treating UDCA non‐responsive patients, the current study was designed to model the interactions between cholangiocytes and CD4+ T cells to investigate potential immunomodulatory mechanisms of bile acid receptor agonists. Human cholangiocytes were co‐cultured with CD4+ T cells to model the biliary stress response. Senescent cholangiocytes were able to polarize T cells toward a Th17 phenotype and suppressed expression of FoxP3 (*P* = 0.0043). Whilst FXR and TGR5 receptor agonists were unable directly to alter cholangiocyte cytokine expression, FGF19 was capable of significantly reducing IL‐6 release (*P* = 0.044). Bile acid receptor expression was assessed in PBC patients with well‐characterized responsiveness to UDCA therapy. A reduction in FXR staining was observed in both cholangiocytes and hepatocytes in PBC patients without adequate response to UDCA. Increased IL‐6 expression by senescent cholangiocytes represents a potential mechanism by which biliary damage in PBC could contribute to excessive inflammation.

**Abbreviations**
BDL: bile duct ligation
FFPE: formalin‐fixed paraffin‐embedded
FGF: fibroblast growth factor
FGFR4: fibroblast growth factor receptor 4
FXR: farnesoid X receptor
IKKB: inhibitor of nuclear factor kappa‐B kinase subunit β
OCA: obeticholic acid
PBC: primary biliary cholangitis
PH: partial hepatectomy
PXR: pregnane X receptor
SASP: senescence associated secretory phenotype
TGR5: takeda G protein‐coupled receptor 5
UDCA: ursodeoxycholic acid

## 1 INTRODUCTION

Cholangiocyte senescence has been observed previously in various liver diseases as a response to ongoing damage in the biliary epithelium.^1^, ^2^, ^3^ When senescent, cholangiocytes secrete a milieu of cytokines and chemokines, referred to as the senescence‐associated secretory phenotype (SASP).^3^, ^4^, ^5^ Of note, the SASP of cholangiocytes consists of a number of cytokines and chemokines involved in CD4+ T helper (Th) cell responses. These include IL‐6, IL‐23, CCL20, and TGFβ, which are key cytokines and chemokines involved in the induction and recruitment of Th17 cells.^6^, ^7^, ^8^, ^9^, ^10^, ^11^

In PBC, similar to other autoimmune diseases, studies have reported T cell skewing toward a Th17‐like phenotype, characterized by increased expression of the transcription factor, RORc, and IL‐17 production.^12^ Previously, FoxP3 expression has been reported to be reduced around the affected bile ducts of PBC livers in comparison to other inflammatory liver diseases indicating a potential Treg deficit.^13^ However, a more recent study has reported an increase in Treg levels in PBC around inflamed portal tracts.^14^ As IL‐12 has also been genetically associated with PBC, this study also analyzed Tbet expression to determine whether Th1 responses may also be induced.^15^, ^16^

Previously, our group has demonstrated a link between cholangiocyte senescence and response to therapy with ursodeoxycholic acid (UDCA) in primary biliary cholangitis (PBC), an autoimmune liver disease targeting the bile ducts.^17^ UDCA therapy helps to limit cholangiocyte stress by ultimately changing the composition of the bile acid pool toward a less toxic phenotype, but does not appear to directly alter immune responses.^18^, ^19^

UDCA is given as a high dose therapy and is thought to work by post‐translationally stimulating expression of hepatic export pumps which helps to stabilize detoxification machinery in the small intestine.^20^, ^21^ Conversely, newer therapies are now focusing on stimulation of specific bile acid receptors. The most studied bile acid receptor agonist is obeticholic acid (OCA)—a selective agonist for the farnesoid X receptor (FXR). In clinical trials, OCA has proven to be effective as a second line therapy in patients with an unsatisfactory response to UDCA, termed “non‐responders” (POISE study; NCT01473524) and is now approved for use as a second line therapy in both Europe and America.^22^

As bile acid receptors control the synthesis and flow of bile acids, they have been of interest as targets in cholestatic disease therapy for some time. However, more recent evidence suggests that activation of these receptors might have anti‐inflammatory and hepatoprotective effects. For example, nuclear bile acid receptors, FXR and pregnane X receptor (PXR), are all involved in the regulation of NFκB‐dependent pro‐inflammatory cytokines, most likely due to interactions with the retinoid X receptor (RXR).^23^, ^24^, ^25^, ^26^ To date, while there are many studies investigating links between bile acid receptor signalling and inflammation, there is a significant lack of understanding regarding this relationship in cholangiocytes.

Additionally, it has been suggested that stimulation of FXR may be protective against fibrosis. For example, the administration of FXR agonists can reduce fibrosis in a bile duct ligation (BDL) model in rats via a SHP‐dependent mechanism which prevents α1 (I) collagen synthesis by hepatic stellate cells.^27^ Furthermore, FXR can interact with PPARγ to prevent transdifferentiation of hepatic stellate cells, subsequently causing a reduction in fibrosis.^28^ In contrast, a more recent study has suggested that a loss of FXR has no positive effect on the severity of fibrosis in four separate mouse models of fibrosis and cholestasis and in some cases a lack of FXR may actually be beneficial.^29^ This study suggests that it is unlikely that FXR agonists are directly stimulating FXR present in hepatic stellate cells to increase fibrosis due to their low level of FXR expression. However, a loss of FXR may result in lower biliary pressure, leading to a less fibrotic response in cholestatic models but not in other models of fibrosis. As such, the role of bile acid receptor signalling in cholestatic disease still requires further investigation.

Takeda G‐protein‐coupled receptor (TGR) 5 is a membrane‐associated bile acid receptor present on the surface of cholangiocytes. TGR5 is involved in bile acid circulation and gall bladder refilling but also plays a hepatoprotective role through the maintenance of the HCO_3_^−^ umbrella.^30^ TGR5 is also capable of inhibiting NFκB signalling through other mechanisms which involve IκB.^31^

Recently, fibroblast growth factor (FGF) 19 and its receptor, FGFR4, have been postulated as potential therapeutic targets. FGF19 (FGF15 in mice) is released in response to activation of FXR and is one of two feedback mechanisms which exists to regulate primary bile acid synthesis.^32^ Previously, FGF19 has been shown to be elevated in the serum of PBC patients and is significantly higher in patients who do not respond to UDCA.^33^ In addition to the ability to reduce the amount of primary bile acids produced by the liver, FGF19 is considered to have anti‐inflammatory and other protective effects.^34^, ^35^, ^36^ The introduction of Fgf15 to *FGF15* ^‐/‐^ mice is able to prevent mortality following partial hepatectomy (PH) and additional FGF15 administration improves liver regeneration in FGF15^+/+^ mice undergoing PH.^34^ Additionally, there is evidence to suggest that activation of FGF receptor 4 (FGFR4), the receptor for FGF19, is able to negatively regulate NFκB via an interaction between FGFR4 and IKKβ.^35^

As the cholangiocyte SASP has been previously linked to UDCA non‐response, we hypothesized that cytokines, such as IL‐6 and CCL20, may be increasing Th17 cell frequencies in these patients. Thus, the current study was designed to determine whether senescent cholangiocytes are capable of altering T cell polarization in vitro. Furthermore, as patients who do not respond to UDCA respond well to FXR agonists, the study also investigated the role of bile acid receptor agonists in this interaction.

## 2 MATERIALS AND METHODS

### 2.1 Cholangiocyte cell culture

The immortalized human intrahepatic cholangiocyte line, H69, was created by and obtained from Grubman and cultured as described.^37^ Cholangiocytes were seeded in 6‐well plates and grown for 24‐72 hours in phenol red‐free media until confluent then stimulated with 200 µmol/L H_2_O_2_ for 2 hours. H69 were then washed in PBS then fresh media was added containing either 10 µmol/L FXR specific agonist, (obeticholic acid; OCA/INT‐747), TGR5/FXR dual agonist (6α‐ethyl‐3α,7α,23‐trihydroxy‐24‐nor‐5β‐cholan‐23‐sulfate sodium salt; INT‐767), TGR5 specific agonist (6α‐ethyl‐23(S)‐methyl‐3α,7α,12α‐trihydroxy‐5β‐cholan‐24‐oic acid; INT‐777), or 50 ng/mL recombinant FGF19 (R&D systems, Abingdon, UK) for up to 72 hours. OCA, INT‐767 and INT‐777 were obtained from Intercept Pharmaceuticals (New York, NY).

Experiments were performed using phenol red‐free media. Senescence induction was confirmed by upregulation of p21 mRNA by quantitative real‐time polymerase chain reaction (qPCR) (Figure S1A). Expression of S100A4 was also analyzed to illustrate that the cytokine response of cholangiocytes was not induced by epithelial to mesenchymal transition (Figure S1A).

### 2.2 CD4+ T cell isolation

Peripheral blood mononuclear cells (PBMC) were isolated from the peripheral blood of healthy volunteers. Blood was diluted at a 1:1 ratio with PBS then layered gently of Lymphoprep density gradient medium (STEMCELL, Cambridge, UK). Samples were centrifuged at 800G for 20 minutes to separate the layers then PBMC were harvested from the interface between the serum and lymphoprep layers. CD4+ cells were then isolated by negative selection using the CD4+ cell isolation kit (Miltenyi, Woking, UK). Purity was assessed by CD4 positivity using immunohistochemistry or flow cytometry (Figure S2).

### 2.3 Cholangiocyte and CD4+ cell co‐culture

**Figure 1:**
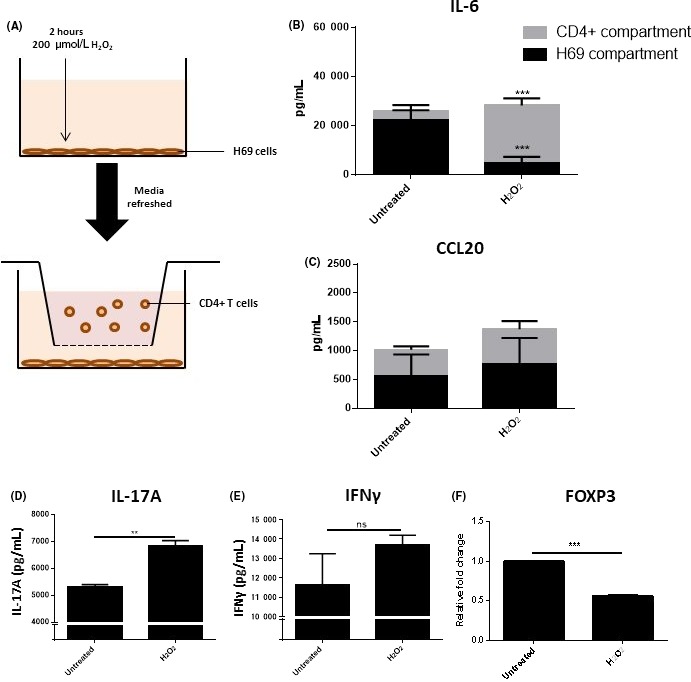
The senescence‐associated secretory phenotype of cholangiocytes pre‐disposes CD4+ cells to a Th17 phenotype. A, Diagram of Transwell co‐culture set up. B and C, Changes in IL‐6 and CCL20 release from H69 cells and CD4+ cells in co‐culture measured by MSD assay. Significance was assessed using two‐way ANOVA (n = 3, ***P < 0.001). D and E, Changes in cytokine release measured by ELISA. (F) FOXP3mRNA expressionmeasured by qPCR in CD4+ cells cultured with either untreated or H2O2‐treated H69 cells. Significance was assessed by paired Student's *t* test (D‐F), **P < 0.01. Graphs are representative of three independent experiments using different T cell donors. Error bars represent standard deviation

Cholangiocytes were seeded at 1 × 10^5^ cells per well in a 24 well companion plate (Corning) in 80 μL phenol red‐free H69 media and cultured overnight. Senescence was induced in cholangiocytes as previously described before addition of CD4+ cells. 0.4 μm inserts (Corning) were placed above with freshly isolated CD4+ cells at a density of 1 × 10^6^ cells in 500 μL Iscove's Modified Dulbecco's Medium (Sigma, Poole, UK) supplemented with 10% FBS, 2 mmol/L L‐glutamine, 100 IU penicillin and 100 μg/mL streptomycin as shown in Figure 1A. CD4+ cells were activated using CD3/CD28 activator beads (Thermo Fisher, Paisley, UK) at a ratio of 1 bead to 2 cells. Co‐cultures were left in culture for 72 hours before analysis.

### 2.4 Patients

Formalin‐fixed paraffin‐embedded (FFPE) human tissue sections from patients with PBC (n = 34) archived at RVI NHS Pathology (Newcastle‐upon‐Tyne, UK). Response to UDCA was assessed using Paris I criteria. Patients with other underlying conditions, such as hepatitis, were excluded from the study. Biopsies taken from healthy livers before transplantation (T0) were used as healthy controls (n = 3). Written informed patient consent was obtained in accordance with research and ethics committee (REC) approval (14/NW/1146).

### 2.5 Immunohistochemistry

For staining of human FFPE liver biopsies, 3 μm sections were dewaxed in xylene for 5 minutes followed by antigen retrieval with citrate buffer in a pressure cooker for 2 minutes. Endogenous peroxidase activity was blocked for 10 minutes in 3% H_2_O_2_ at room temperature. The slides were then washed in TBS for 2 × 5 minutes. Slides were blocked with an AB blocking kit (Vector Laboratories, Peterborough, UK) and stained with either rabbit or mouse VECTASTAIN Elite ABC peroxidase kits (Vector Laboratories) as appropriate. Slides were incubated in primary antibody specific for FXR (R&D systems, 1:50), PXR (GeneTEX, 1:75), TGR5 (Abcam, 1:75), FGFR4 (Abcam, 1:100) or CD4 (Abcam, 1:100) for 1 hour. Colour was developed using DAB substrate. When dual staining, VECTASTAIN Immpress Universal peroxidase kit (Vector laboratories) was used in combination with ImmPACT SG peroxidase substrate (Vector Laboratories). Following staining, slides were dehydrated in 70%‐99% ethanol followed by xylene then mounted in DPX (CellPath, Newtown, UK).

For characterization of FGFR4 on the H69 cell line, untreated/hydrogen peroxide‐treated wells of a chamber slide were incubated for 48 hours and then fixed in methanol for 10 minutes and frozen. Slides were washed in TBS then stained using with VECTASTAIN Immpress Universal peroxidase kit/Elite ABC peroxidase kit (Vector laboratories) according to manufacturer's instructions using FGFR4 (Abcam), p21 (Abcam), TGR5 (Abcam) or FXR (R&D Systems) primary antibodies.

For all staining, a “no primary antibody” slide was used as a negative control. All scoring was performed blinded by two independent assessors at 20× magnification in an area including at least one portal tract. A minimum of five portal tracts were examined per sample. For CD4 and FXR stained sections, staining was quantified using a score‐based system to estimate the amount of staining as the high numbers of positive cells present in many of the sections meant that it was not possible to perform a manual cell count accurately. Sections were scored on a scale of 1‐4 with 1 indicating lowest expression and 4 representing highest expression. Slides were imaged using an Olympus SC50 microscope camera and CellSens Standard imaging software (Olympus, Southend‐on‐Sea, UK).

### 2.6 Quantitative real‐time polymerase chain reaction (qPCR)

RNA was extracted using Qiagen RNEasy kits (Qiagen, Machester, UK) and assessed for purity using a NanoDrop ND‐1000 (Thermo Scientific, Wilmington, DE). cDNA was synthesized using the Bioline Tetro cDNA synthesis kit (Bioline, London, UK). All TaqMan primer/probes were obtained from Thermo Fisher Scientific. The following primers were used in this study: CCL20 (Hs00355476_m1), FOXP3 (Hs01085834_m1), GAPDH (Hs02758991_g1), IL‐1β (Hs00174097_m1), IL‐6 (Hs00985639_m1), SHP (Hs00222677_m1). Primer information is detailed further in the supplementary material. Each reaction was run for 40 cycles on a StepOnePlus real‐time PCR machine (Thermo Fisher Scientific) using SensiFAST probe Hi ROX kit (Bioline).

### 2.7 ELISA

Cholangiocytes were cultured and stimulated with FGF19, OCA, INT‐767 or INT‐777 as previously described. Supernatants were centrifuged at 6000G for 5 minutes before use and stored at −80°C prior to use. Concentrations of IL‐6 and IL‐17A were assayed using DuoSet ELISA kits (R&D systems) according to manufacturer's instructions.

### 2.8 MSD

Multiplex cytokine analysis of co‐culture models was assessed using a custom 96 well U‐PLEX panel (Meso Scale Discovery, Rockville, MD) according to manufacturer's instructions. The panel included IFNg, IL‐17A, IL‐17F, IL‐12p70, IL‐1β, IL‐6, IL‐10, CCL2 and CCL20. In addition, samples were assayed on the TGFβ U‐PLEX panel (Meso Scale Discovery) containing antibodies for TGFβ1, TGFβ2 and TGFβ3.

## 3 RESULTS

### 3.1 The biliary SASP influences CD4+ cell polarization toward a Th17 phenotype

To address the question of whether the stressed biliary epithelium was capable of altering T cell polarization we developed a transwell co‐culture model containing H69 cells in the lower chamber and CD4+ T cells from healthy volunteers in the upper chamber (Figure 1A). We have previously determined that the H69 cell line secretes a similar cytokine profile when senescent to that described by primary cholangiocytes (Figure S3). Seventytwo hours after induction of senescence in cholangiocytes, IL‐6 protein secretion was significantly increased in the upper chamber of the Transwell system containing the CD4+ cells in comparison to controls (Figure 1B; *P* ≤ 0.001). In comparison, levels of IL‐6 in the H69 compartment were reduced, potentially suggesting an uptake of IL‐6 production by the CD4+ cells. Similary, CCL20 secretion was not significantly altered in either compartments (Figure 1C). This may also be reflective of uptake from CD4+ cells. IL‐1β expression was not detectable by ELISA and was only detectable at very low levels by MSD (data not shown).

Analysis of the T cell component suggested skewing toward a Th17‐like phenotype. Consistent with this, IL‐17A protein release was increased in wells of CD4+ cells exposed to senescent H69 (Figure 1D). IFNγ expression was not significantly altered but showed a trend toward increased expression following the induction of senescence (Figure 1E). Expression of FOXP3 was significantly downregulated in CD4+ cells co‐cultured with senescent cholangiocytes in comparison to those cultured with unstimulated cholangiocytes (Figure 1F). Experiments with conditioned media showed no significant change in FOXP3, Tbet or RORC expression when cultured with CD4+ T cells (Figure S4).

### 3.2 FXR and TGR5 agonists do not influence cytokine production in a model of biliary damage

**Figure 2:**
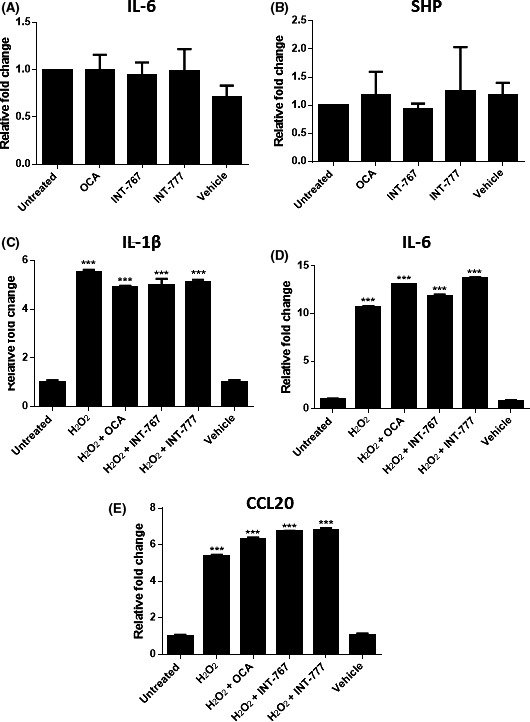
Response of the H69 cell line to FXR and TGR5 activation. A and B, Changes in IL‐6 and SHP mRNA expression in the H69 cell line following treatment with 10 μmol/L OCA, INT‐767 or INT‐777 after 24 hours. 0.001% Ethanol was used as a vehicle control. C‐E, Changes in mRNA expression of IL‐1β, IL‐6 and CCL20, respectively, following senescence induction. Significance was assessed by one‐way ANOVA relative to untreated control, *P < 0.05. Graphs are representative of three independent experiments. Error bars represent standard error

Following the establishment of a model of cytokine‐mediated interactions between CD4+ cells and cholangiocytes, a series of experiments was performed to investigate potential mechanisms to modulate these interactions. As FXR agonists have shown the potential to improve clinical status in patients shown not to respond to conventional therapy with UDCA, it was hypothesized that bile acid receptor agonism might modulate cytokine release from the biliary epithelium thereby altering the inflammatory response. Data from these studies indicate that treatment of cholangiocytes with either FXR or TGR5 agonists was not sufficient to alter this response (Figure 2). No effect on IL‐6 or SHP expression was observed in non‐senescent cholangiocytes following the addition of bile acid receptor agonists (Figure 2A,B). SHP expression has been reported to increase following stimulation of FXR and was consequently included to determine whether these pathways were activated in cholangiocytes.^38^ Similarly, there was also no decrease in IL‐1β, IL‐6 or CCL20 mRNA expression following addition of bile acid receptor agonists in the senescent cholangiocytes (Figure 2C‐E). Expression of FXR in the H69 cell line was only detectable at very low levels, indicating that the lack of response to FXR agonists may be due to a lack of FXR expression (Figure S3A,B). Both OCA and INT‐767 were capable of FXR activation in the HepG2 cell line as indicated by SHP expression (Figure S5A). A reduction in pERK was seen in H69 cells following activation with either INT‐767 or INT‐777 suggesting some activation of TGR5 signalling following stimulation with TGR5 agonists (Figure S5B).

### 3.3 FXR expression is decreased in UDCA ‘non‐responders’

**Figure 3:**
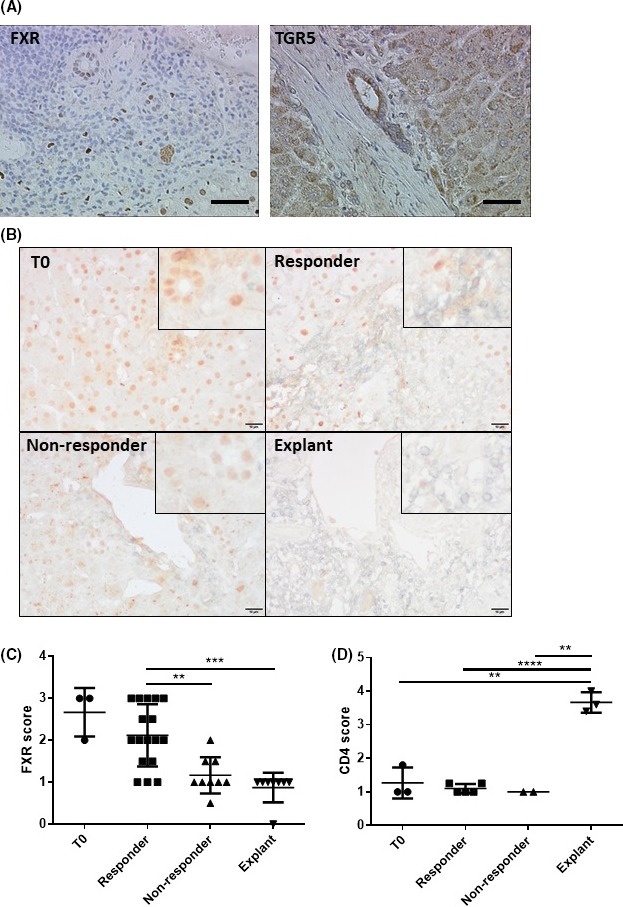
Bile acid receptor expression in biopsies and explants from PBC patients. A, Representative images of FXR and TGR5 staining in PBC patients. Scale bars represent 50 μm. B, Representative images of FXR (brown) and CD4 (silver) stains from T0, responders, non‐responders and explants, respectively. Scale bars represent 10 μm. C and D, FXR and CD4 expression in PBC biopsies and explants were scored from 1 (low) to 4 (high). C, Bars represent the mean of at least 8 different cases per PBC cohort and 3T = 0 cases. D, Bars represent the mean of at least two different cases per group and 3T = 0 cases. Significance was measured by one‐way ANOVA. **P < 0.01

Figure 3A shows general expression of bile acid receptors in PBC liver. FXR expression was typically expressed in the nuclei of hepatocytes and the biliary epithelium with some expression also seen in infiltrating inflammatory cells. In contrast, TGR5 expression was more restricted to the apical surface of cholangiocytes. It was noted that FXR expression was reduced in patients identified as UDCA “non‐responders” (Figure 3B). This loss of receptor expression did not appear to be related to levels of CD4+ cell infiltration and was identified in both early stage biopsies and late stage explants from “non‐responder” patients (Figure 3C‐D). Negative control slides are shown in Figure S6. Interestingly, the FXR present in hepatocytes of the “non‐responder” cohort was typically cytoplasmic in comparison with the punctate nuclear staining seen in the hepatocytes and bile ducts in both healthy control livers and responders to UDCA therapy. No significant change in PXR or TGR5 expression was observed in PBC patients with regard to either levels of CD4+ inflammation or UDCA response status (data not shown).

### 3.4 FGF19 is capable of reducing IL‐6 release from H69 cells

**Figure 4:**
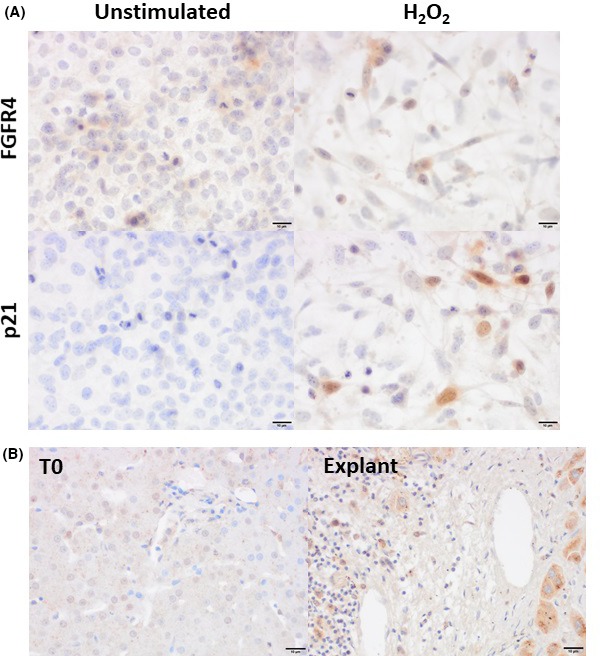
Expression of FGFR4 in cholangiocytes following senescence induction. A, Representative images of H69 cells cultured for 48 hours following senescence induction with 200 μmol/L H_2_O_2_. B, Representative staining of FGFR4 in healthy control (T0) tissue and PBC explants. Scale bar represents 10 μm

Clinical trials have indicated that patients who do not respond to UDCA do show a positive response to therapy with FXR agonists. Thus, it was hypothesized that FXR signalling may only be reduced in the liver and may still function normally in other organs, such as the intestines. As FGF19 is released downstream of FXR signalling and circulated back to the liver through the portal blood we investigated whether FGF19 may be capable of modulating signalling in the H69 cell line. FGFR4 was expressed in unstimulated cholangiocytes and expression was maintained following induction of senescence, illustrated by induction of p21 expression (Figure 4A). Furthermore, FGFR4 expression could also be seen clearly in PBC liver explants (Figure 4B) contrasting with minimal expression in healthy control (T0) healthy biopsy tissue.

**Figure 5:**
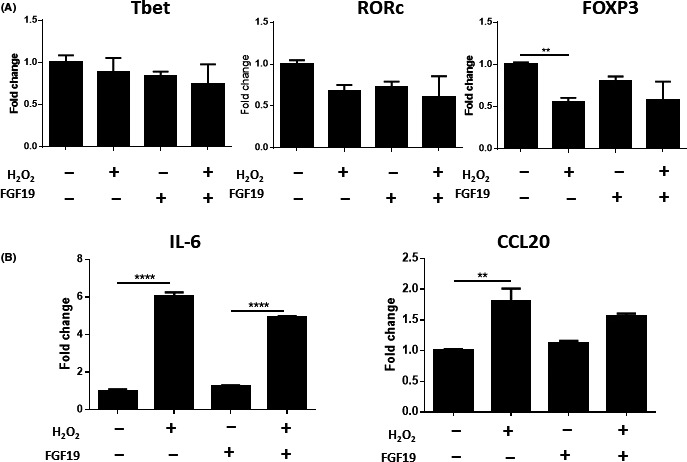
Effects of FGF19 on co‐cultured H69 and CD4+ cells. A, Changes in mRNA expression in CD4+ cells cultured with H69. B, Changes in mRNA expression in H69 cells co‐cultured with CD4+ cells. Significance was assessed using one‐way ANOVA, *P* < 0.001, n = 3 (**)

While the addition of FGF19 showed little change in cytokine mRNA expression in senescent cholangiocytes, the addition of FGF19 was capable of reducing IL‐6 protein expression (*P* < 0.006) to levels comparable with untreated H69 cells (Figure 5A,B)Addition of FGF19 into the co‐culture system was not sufficient to cause a recovery of FOXP3 expression in CD4+ cells (Figure 5A). Furthermore, FGF19 did not cause a consistent reduction in IL‐6 mRNA expression (Figure 5B). There was also no significant decrease in CCL20 expression between senescent cholangiocytes and senescent cholangiocytes treated with FGF19 (Figure 5B).

**Figure 6:**
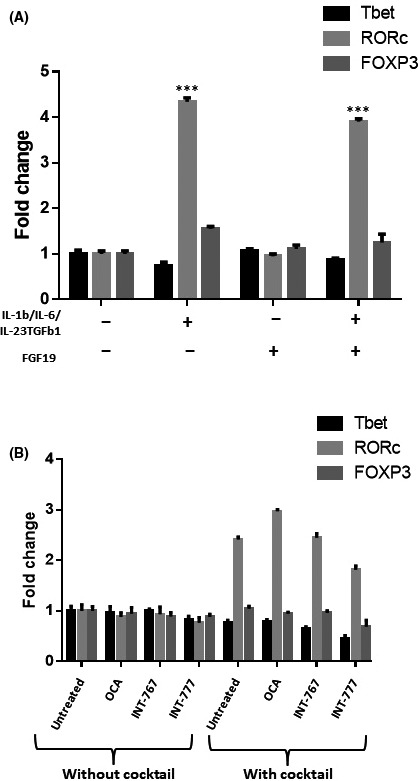
Effects of FGF19 and bile acid receptors on Th17 cell polarization. A, Effects of FGF19 on Th17 polarization. CD4+ cells activated with CD3/CD28 beads were cultured with IL‐1β, IL‐6, IL‐23 and TGFβ1 to induce Th17 polarization then cells were cultured with or without FGF19 (50 ng/mL; A), INT‐747, INT‐767 or INT‐777 (10 μmol/L; B) for 72 h. Significance was assessed using one‐way ANOVA, *P* < 0.001 (**). Graphs are representative of three independent experiments and error bars represent standard error

There was also no evidence to suggest that either FGF19 or FXR and TGR5 agonists were capable of altering T cell polarization directly (Figure 6). CD4+ T cells were artificially polarized toward a Th17 phenotype using a polarizing cocktail of cytokines with or without the addition of FGF19 or bile acid receptor agonists. Neither FGF19 nor any of the bile acid receptor agonists prevented the induction of RORc in the CD4+ T cells (Figure 6A,B). No consistent change in Tbet or FOXP3 expression was determined in any of the samples.

## 4 DISCUSSION

The data presented in this paper show that the biliary SASP may be contributing to the pathogenic T cell phenotype reported in PBC patients. Immunohistochemistry on PBC patients has previously demonstrated release of cytokines typically associated with Th17 cells, specifically IL‐6, IL‐23, and CCL20.^1^, ^5^, ^12^ We found that many of these cytokines were up‐regulated by the cholangiocytes following senescence induction, although IL‐6 was by far the most abundant. Previous in vitro experiments have demonstrated that IL‐6 is essential for Th17 polarization in humans.^6^, ^8^ Blockade of IL‐6 has proven useful in the treatment of rheumatoid arthritis, another T cell‐mediated autoimmune disease, and therapeutic agents which target this pathway may also be beneficial in PBC.^39^ Our data also suggest that CCL20 could play a role in PBC pathogenesis. However, while CCL20 production was increased in senescent cholangiocytes, these levels were not altered by treatment with either bile acid receptor agonist or FGF19. Although H69 cells undergoing senescence did appear to upregulate IL‐1β mRNA, very little or no IL‐1β protein was detectable by MSD or ELISA analysis, suggesting that this cytokine is unlikely to play a significant role in this model.

We also observed that senescent cholangiocytes were able to reduce FoxP3 expression in CD4+ T cells in addition to causing an increase in IL‐17 production, suggesting a potential shift toward a Th17 phenotype. However, while IL‐17 expression was increased, this was not accompanied by an increase in RORc expression. This suggests that, in this model, the increased IL‐17 production may arise due to a lack of suppression of the existing IL‐17‐producing T cells by Treg. While conditioned media was not capable of causing a significant difference in either FOXP3 or Tbet expression, this may be explained by the extended length of culture needed for T cell polarization to take place. It may be the case that the cytokines needed for polarization were not stable for 72 hours after the addition of conditioned media, and that a constant production of cytokine from the presence of cholangiocytes is necessary to produce the levels required to induce this response.

We found that FXR and TGR5 agonists were unable to abrogate the release of pro‐inflammatory cytokines in our model at concentrations comparable to those used in previous studies.^40^, ^41^ The cholangiocyte line expressed low levels of FXR in comparison to cholangiocytes within healthy liver, but still retained levels of TGR5 (Figure S3). This is characteristic of cholangiocytes from patients who do not respond to therapy with UDCA. While there was no obvious increase in TGR5 expression between responders and non‐responders, TGR5 expression was shown to increase in response to oxidative stress of the cholangiocyte line (Figure S3D). TGR5 expression then reduces back toward normal level after 48. Furthermore, other studies have suggested that TGR5 has a protective effect in maintenance of the HCO_3_^‐^ “umbrella.”^30^ Therefore, while TGR5 may not influence cytokine release by the cholangiocyte SASP, there is evidence to suggest that TGR5 agonists may be beneficial in maintenance of bile flow and protection against toxic concentrations of bile acids.

As UDCA “non‐responders” show improvement following treatment with OCA, FXR signalling must still be functional in some capacity.^42^ While a decrease was observed in FXR expression in the livers of PBC patients, intestinal FXR expression may still function normally. Thus, release of FGF19 represents a potential mechanism by which FXR signalling may be affecting the liver in these patients. A significant effect on T cell polarization was not observed in the co‐culture, model the release of FGF19 into the liver would likely still have some beneficial effects in reducing bile acid production. Additionally, there is some evidence that FGF19 may have regenerative roles within the liver.^34^ Recent evidence suggests that the FGF19 feedback mechanism is still intact in PBC patients and FGF19 analogues are currently being studied as potential therapeutic agents.^36^, ^43^ The FGF19 analogue, NGM282, is currently in phase II clinical trials (NCT02026401).

It is also important to note that, while no significant change in T cell polarization was observed, the effects of FXR or TGR5 agonism on other immune cell types within this system were not studied. TGR5 is expressed on other immune cells in the liver, and has been particularly well studied in Kupffer cells.^44^, ^45^ As Kupffer cells also produce many cytokines which polarize T cells, including IL‐6, and have also been linked to bile acid receptor signalling this cannot be discounted as a potential therapeutic mechanism.^46^, ^47^, ^48^ It is also possible that FXR and TGR5 agonists may have a beneficial role in PBC patients through other hepatoprotective mechanisms, such as reduction of fibrosis or increased HCO_3_^−^ output.^27^, ^30^ However, the long‐term benefits of bile acid receptor agonism on human liver histology remain unknown and require further study.

In summary, the results from this study suggest that FXR agonists have a capacity to regulate cytokine release via FGF19, although this may not be sufficient alone to alter T cell polarization. Although further research is required to fully understand the complex and dynamic interactions which occur in vivo, these findings go some way toward improving our understanding of the dynamic relationships which occur between the human immune system and the biliary microenvironment in PBC.

5

**Figure 7:**
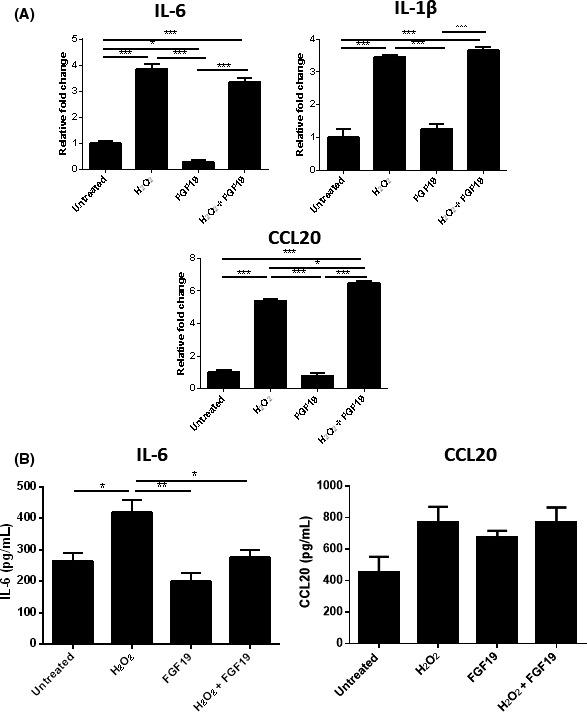
Response of senescent H69 cholangiocytes to 50 ng/mL FGF19. A, Expression of FGFR4 (brown) in the H69 cell line (left) and in a bile duct from a PBC explant liver (right). Scale bars represent 30 μm (left panel) and 50 μm (right panel). B, Changes in mRNA expression of Th17 associated cytokines and chemokines in stressed H69 in response to FGF19. C, Changes in IL‐6 and CCL20 release by stressed H69 in response to FGF19 measured by ELISA. Significance was assessed by one‐way ANOVA. *P < 0.05. Graphs are representative of three independent experiments and error bars represent standard deviation

## CONFLICT OF INTEREST

The authors have no disclosures to make.
