# Supplementary figures and images for: Bile acid receptor agonists in primary biliary cholangitis: Regulation of the cholangiocyte secretome and downstream T cell differentiation
Source: FASEB Bioadv. 2019 Apr 22;1(5):332–43. doi: 10.1096/fba.2018-00046 (PMC6996327; doi:10.1096/fba.2018-00046)

**A****p21****24h**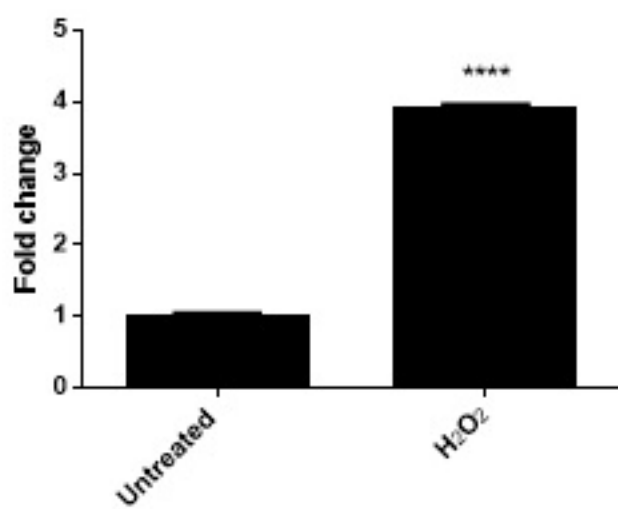**48h**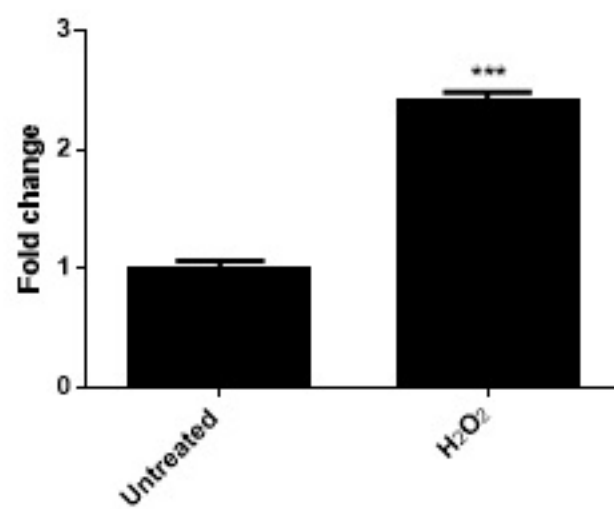**72h**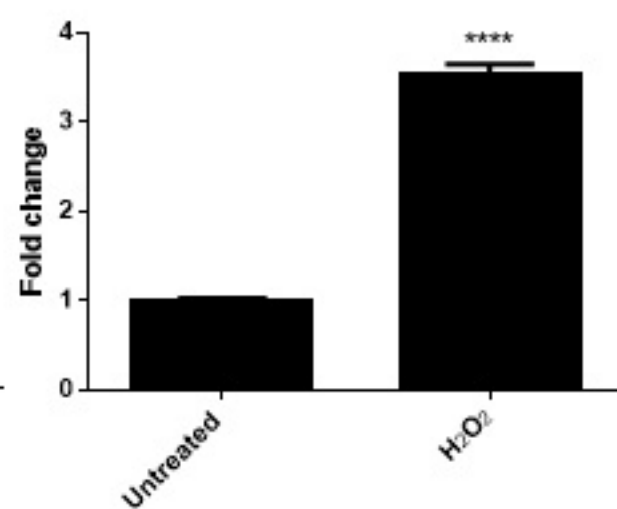**B****S100A4****24h**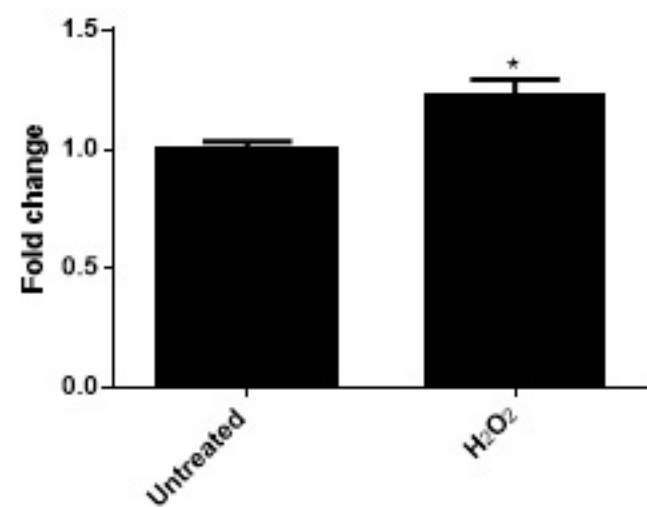**48h**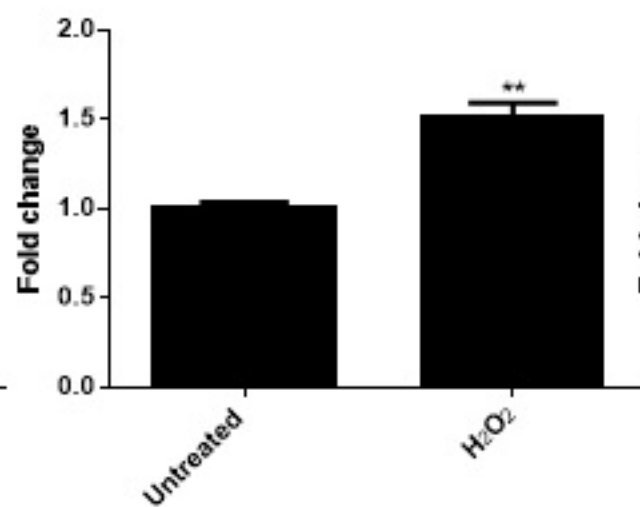**72h**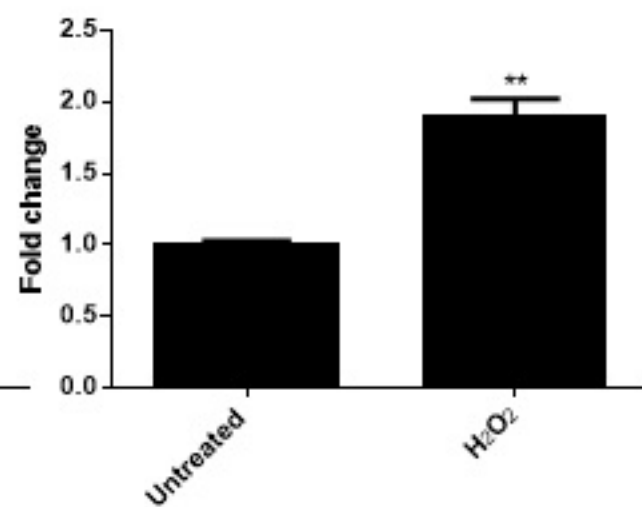

Supplement: Supplementary file 1 [file FBA2-1-332-s001.zip › fba21044-sup-0001-FigS1.jpg]

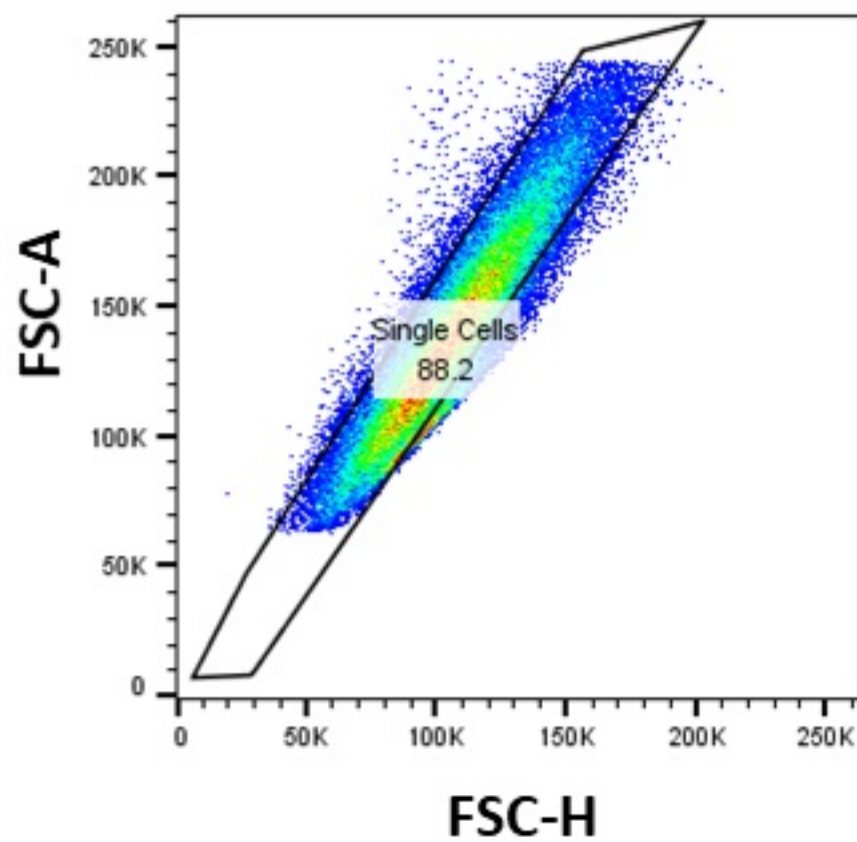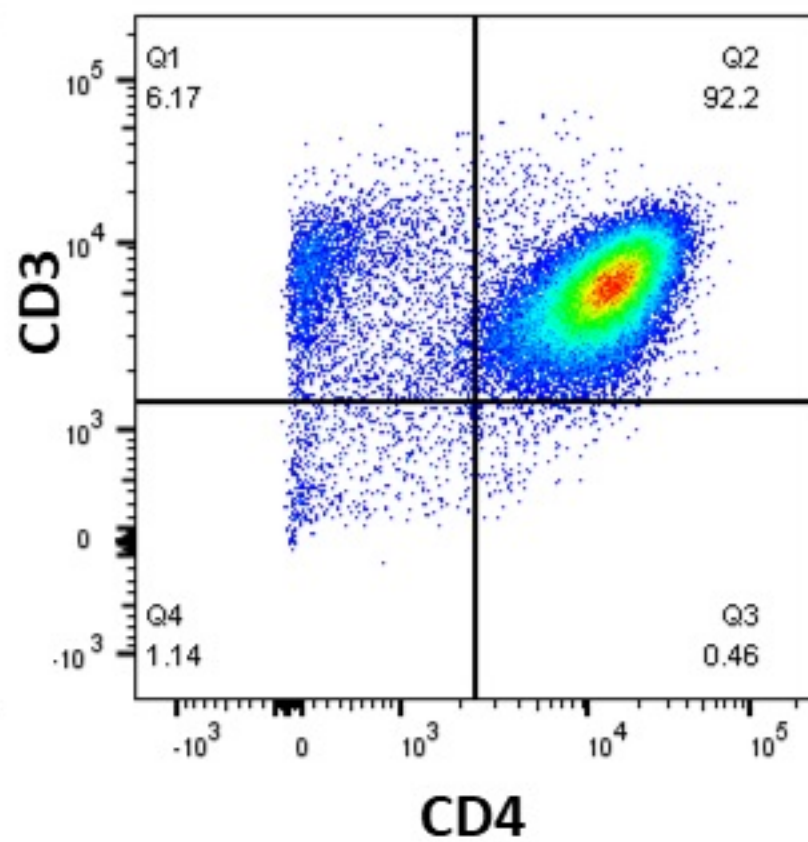

Supplement: Supplementary file 1 [file FBA2-1-332-s001.zip › fba21044-sup-0002-FigS2.jpg]

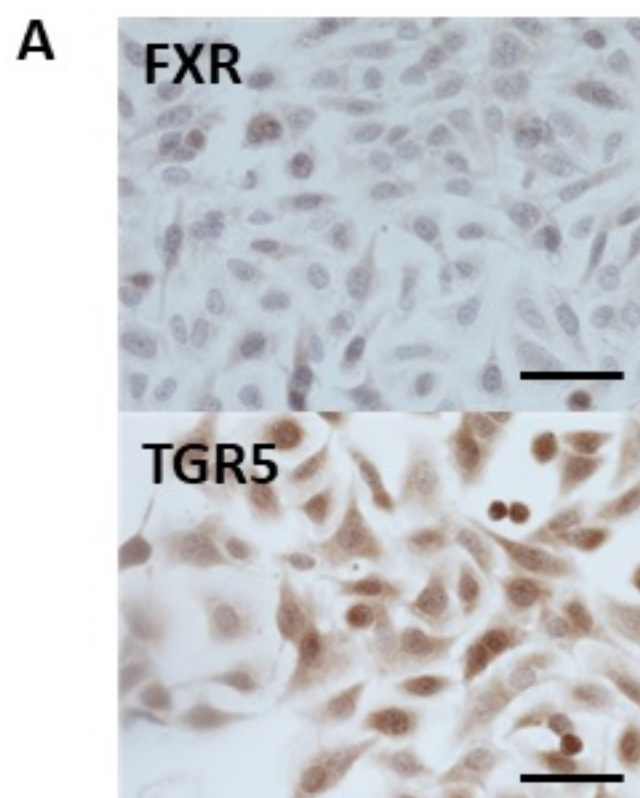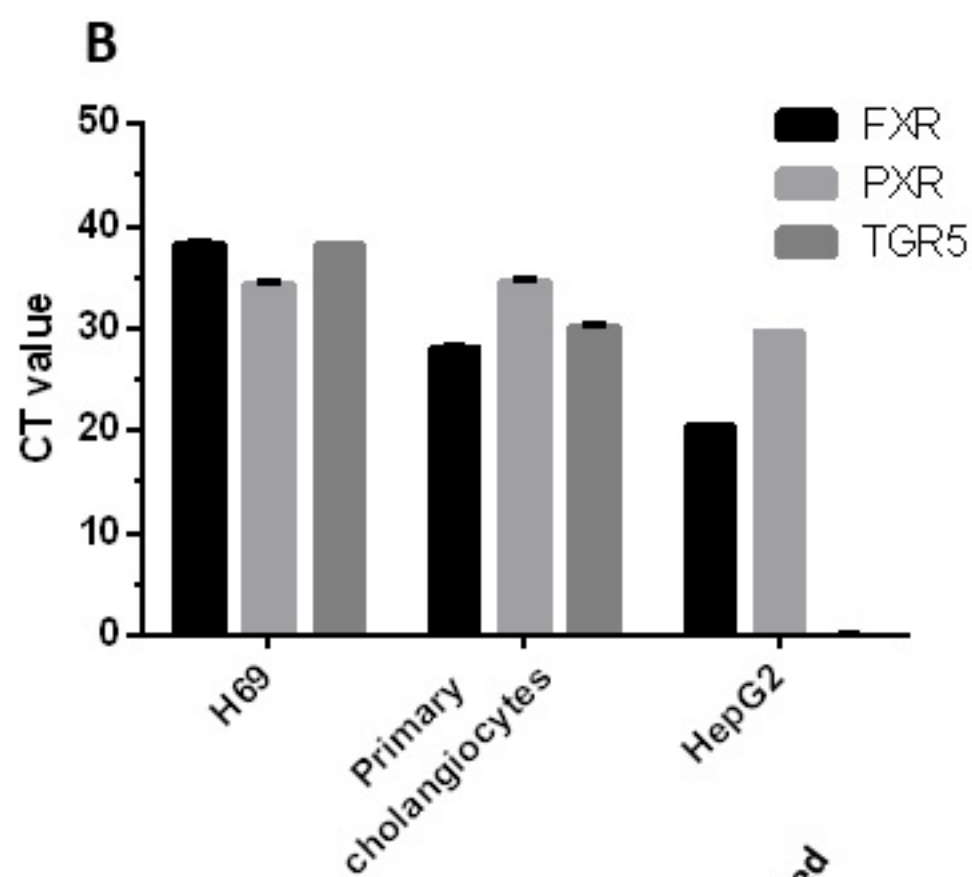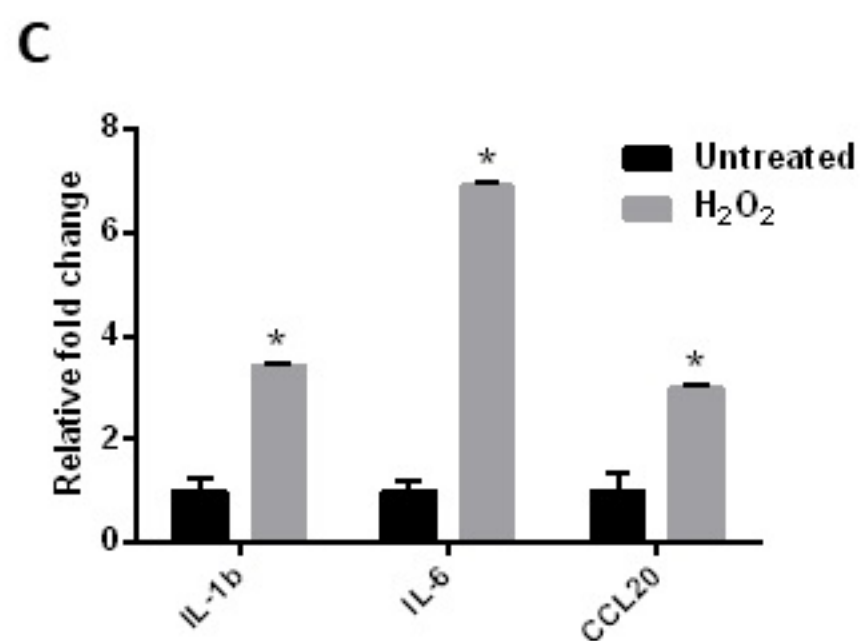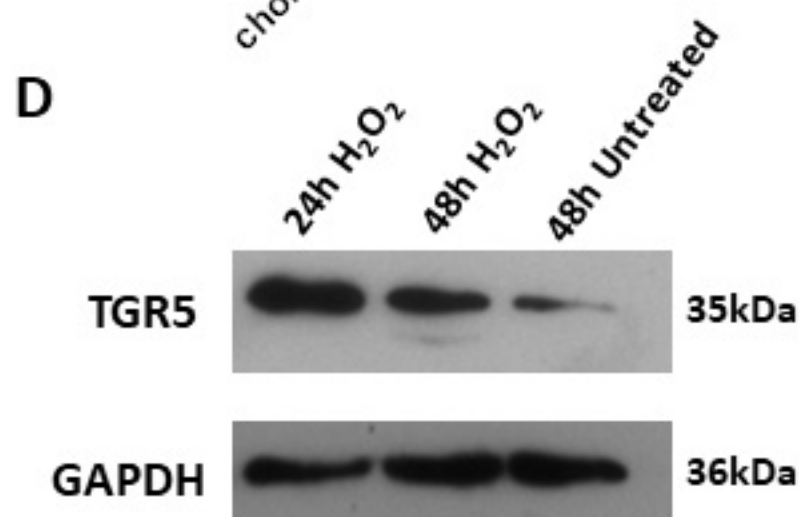

Supplement: Supplementary file 1 [file FBA2-1-332-s001.zip › fba21044-sup-0003-FigS3.jpg]

### FOXP3

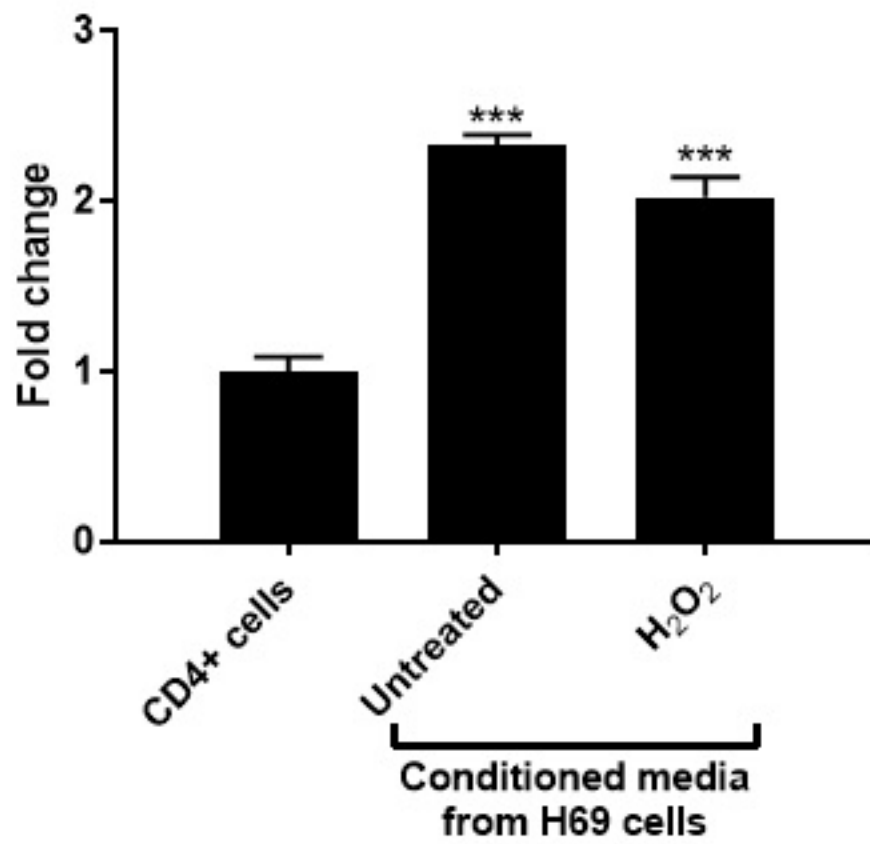

### RORc

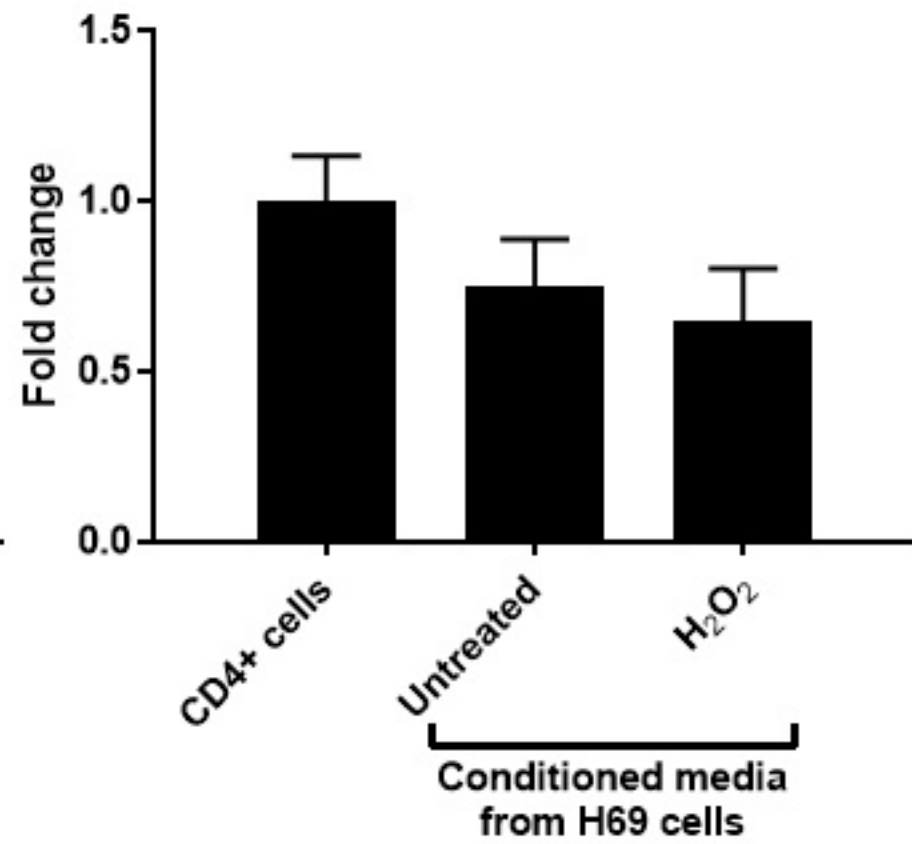

### Tbet

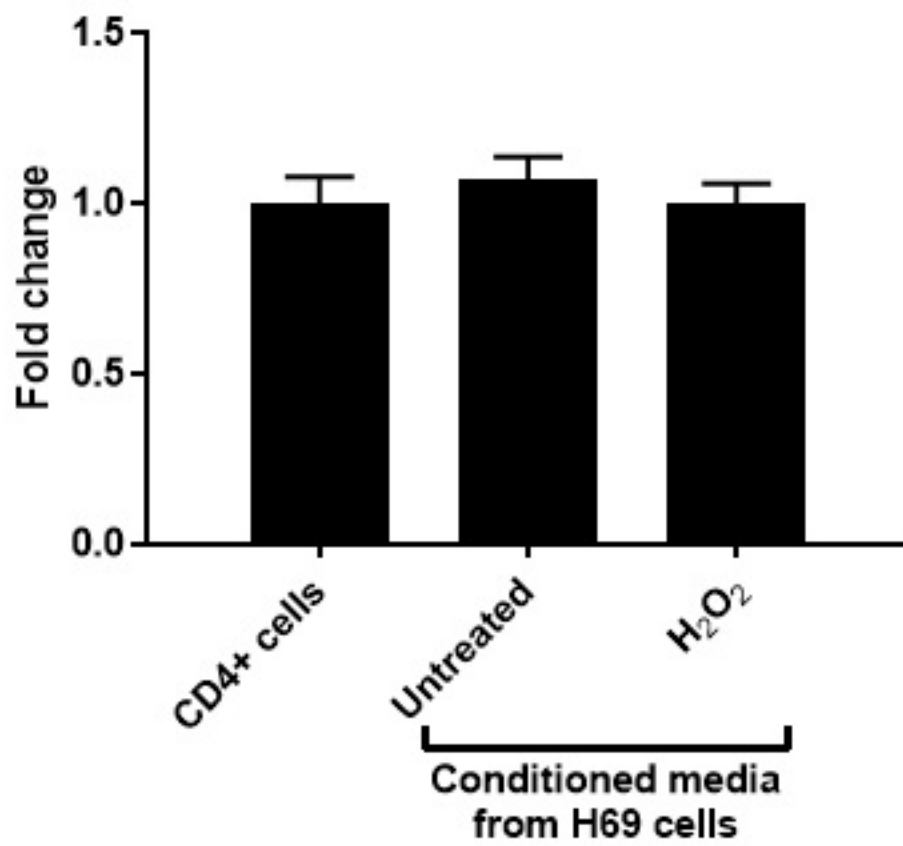

Supplement: Supplementary file 1 [file FBA2-1-332-s001.zip › fba21044-sup-0004-FigS4.jpg]

A

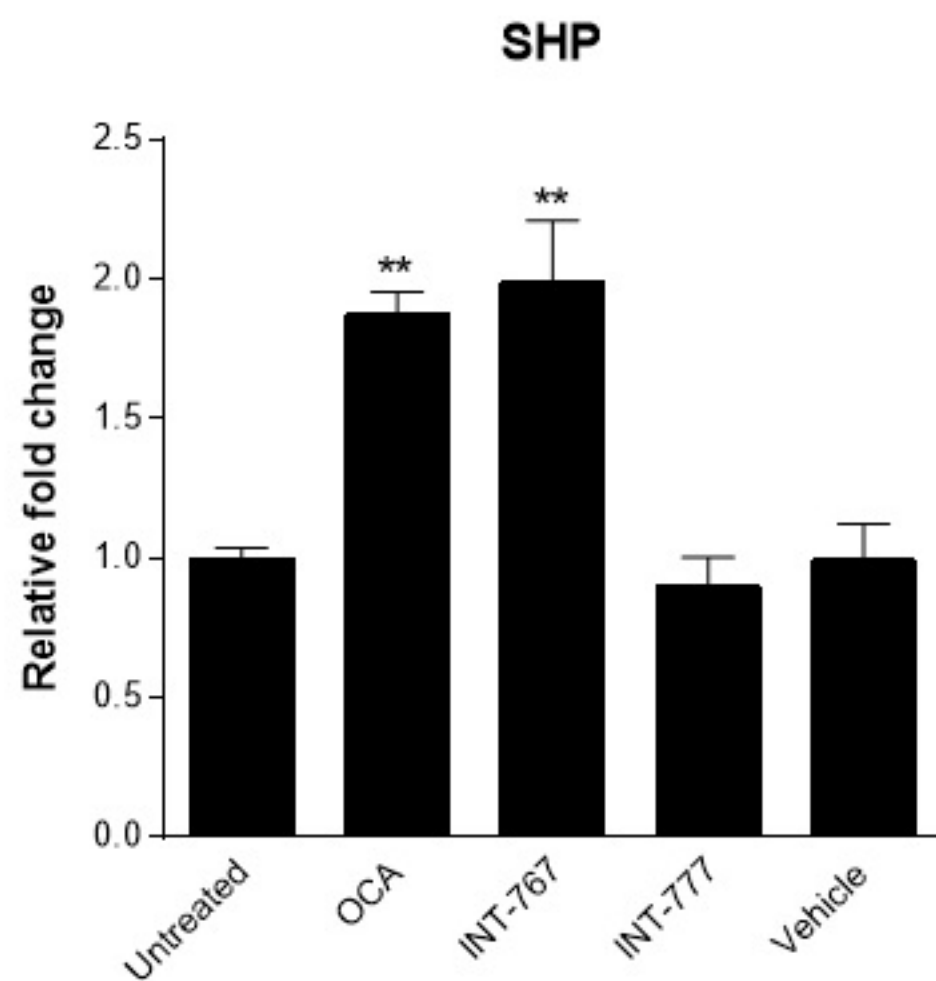

B

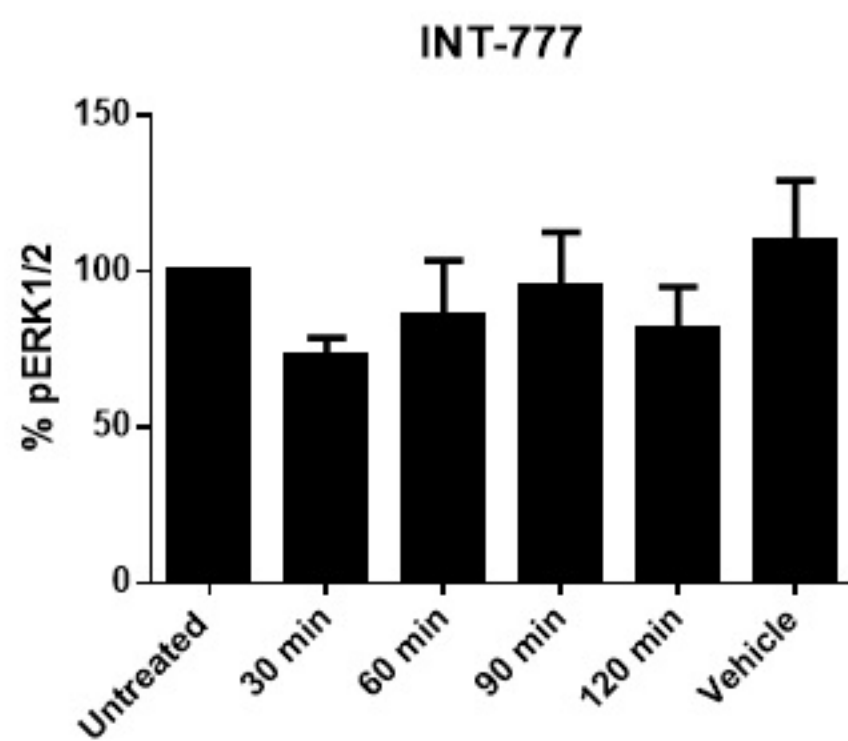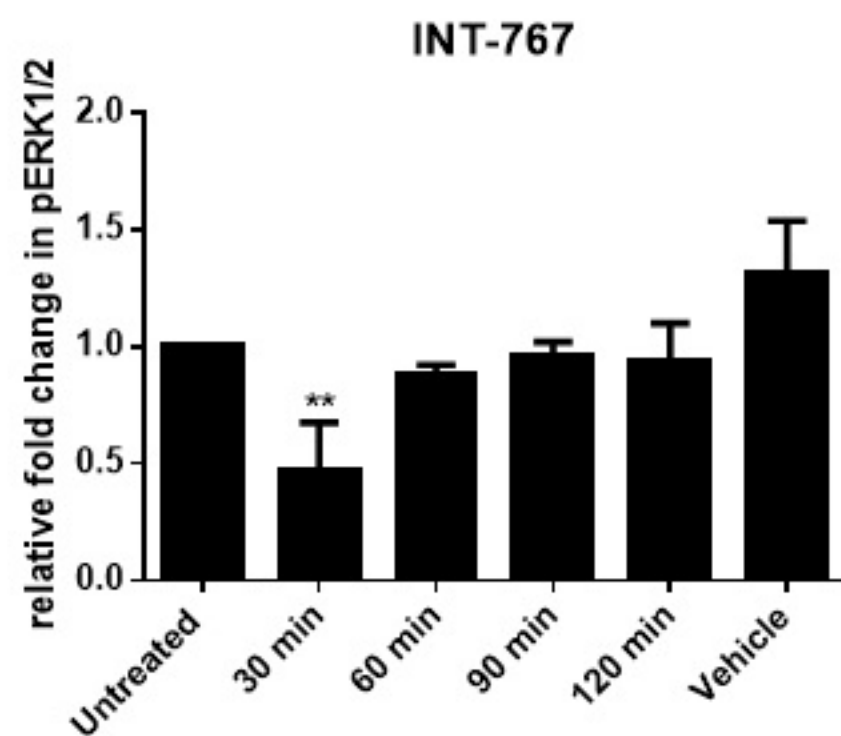

Supplement: Supplementary file 1 [file FBA2-1-332-s001.zip › fba21044-sup-0005-FigS5.jpg]

**A**

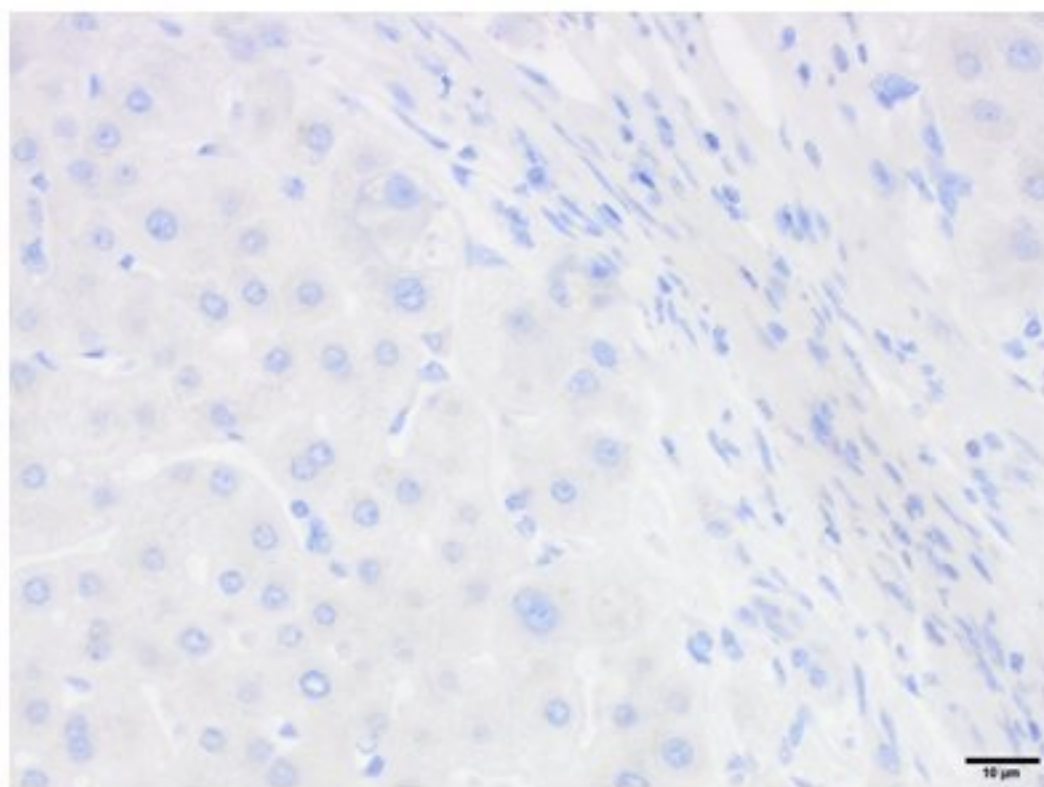

**B**

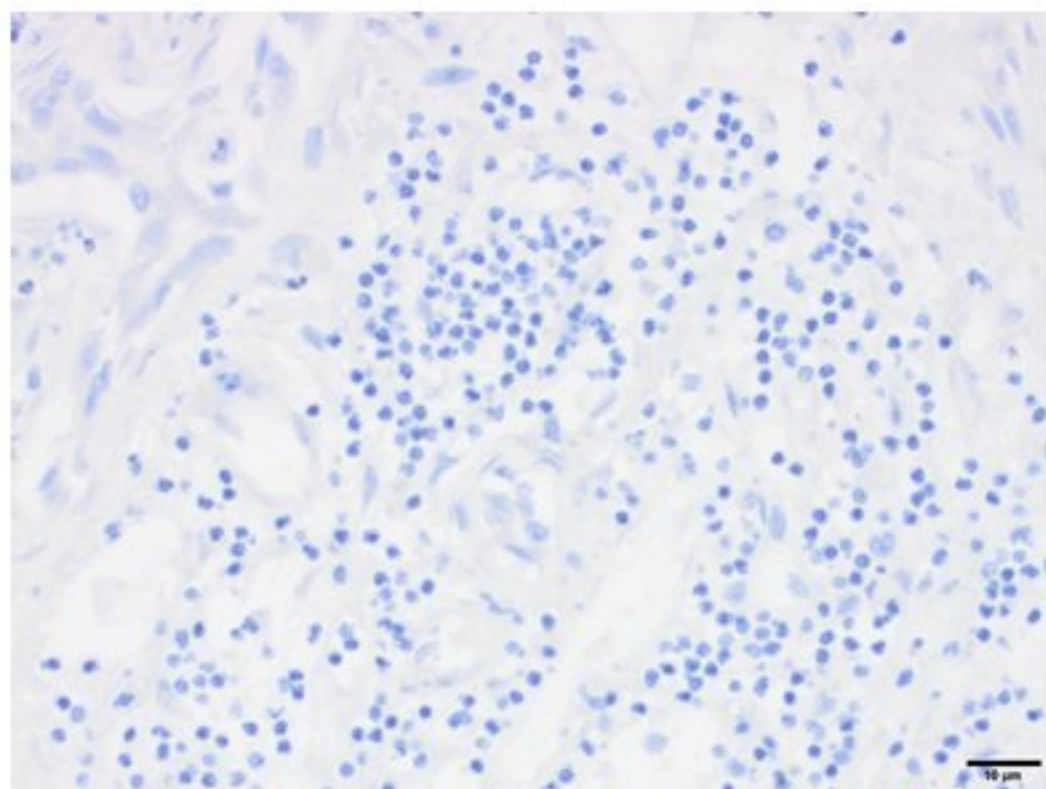

Supplement: Supplementary file 1 [file FBA2-1-332-s001.zip › fba21044-sup-0006-FigS6.jpg]

**A**

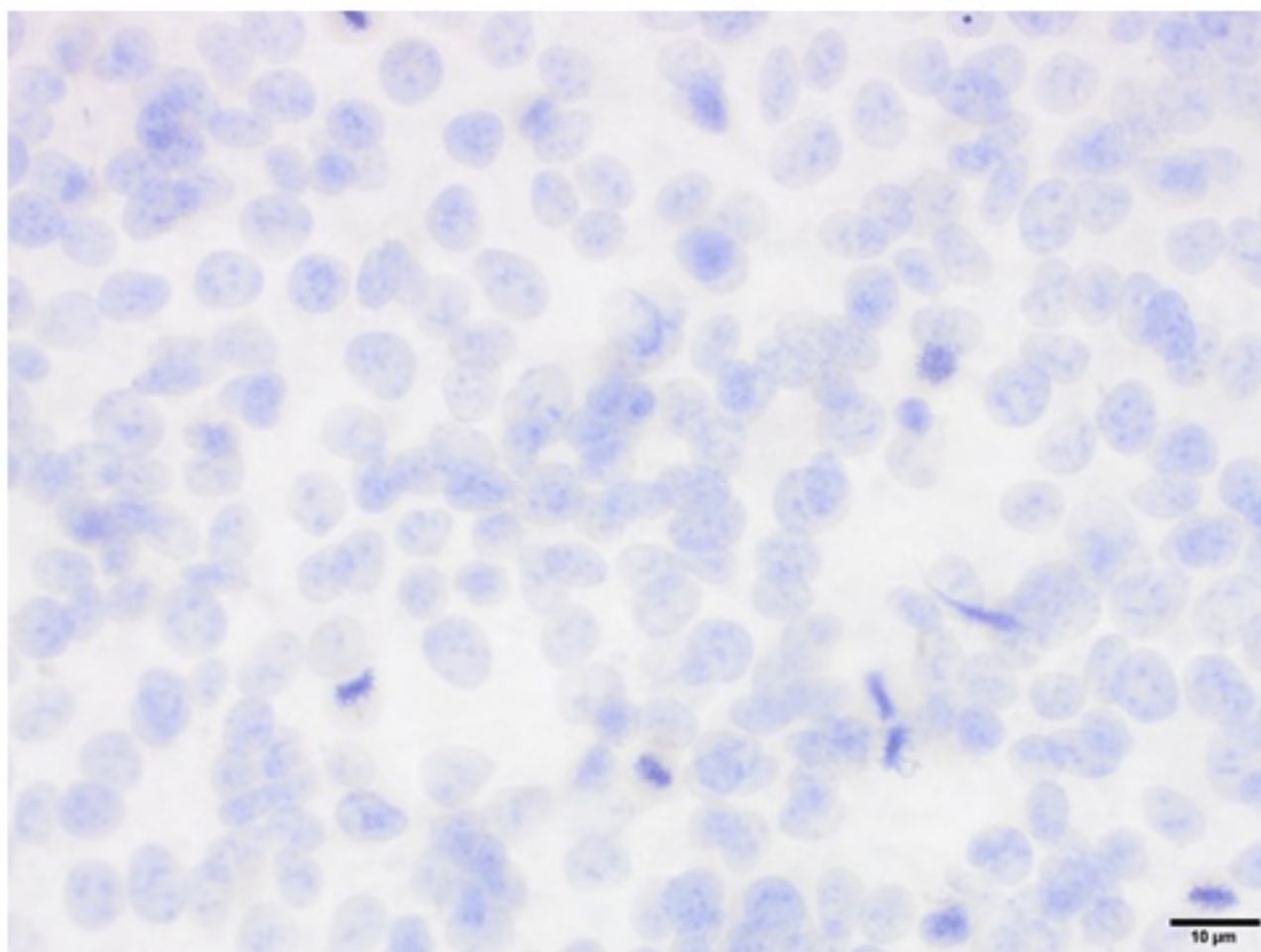

**B**

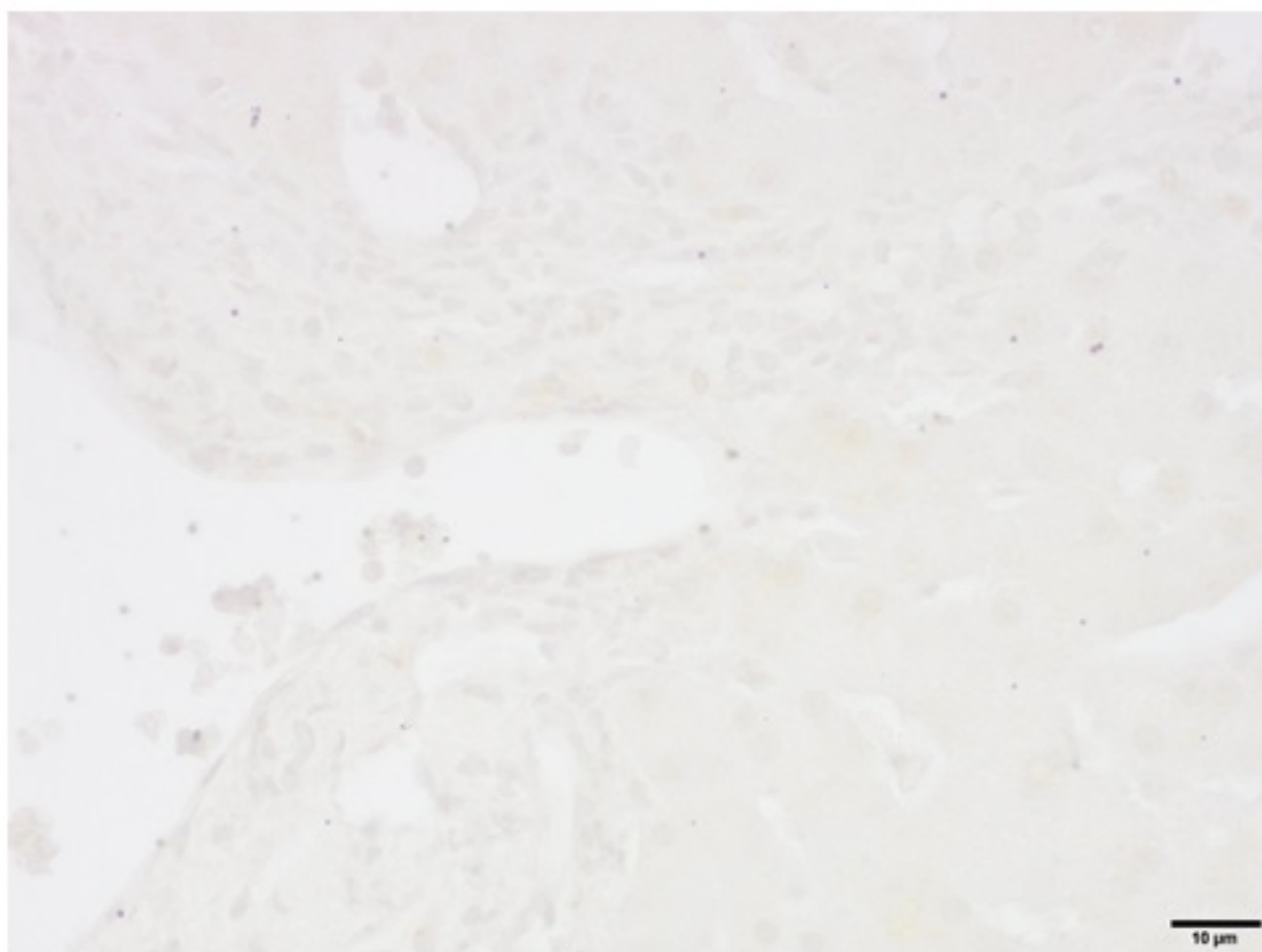

Supplement: Supplementary file 1 [file FBA2-1-332-s001.zip › fba21044-sup-0007-FigS7.jpg]
